# ePhenotyping for Abdominal Aortic Aneurysm in the Electronic Medical Records and Genomics (eMERGE) Network: Algorithm Development and Konstanz Information Miner Workflow

**Published:** 2015-07-30

**Authors:** Kenneth M Borthwick, Diane T Smelser, Jonathan A Bock, James R Elmore, Evan J Ryer, Zi Ye, Jennifer A. Pacheco, David S. Carrell, Michael Michalkiewicz, William K Thompson, Jyotishman Pathak, Suzette J Bielinski, Joshua C Denny, James G Linneman, Peggy L Peissig, Abel N Kho, Omri Gottesman, Harpreet Parmar, Iftikhar J Kullo, Catherine A McCarty, Erwin P Böttinger, Eric B Larson, Gail P Jarvik, John B Harley, Tanvir Bajwa, David P Franklin, David J Carey, Helena Kuivaniemi, Gerard Tromp

**Affiliations:** 1 The Sigfried and Janet Weis Center for Research, Geisinger Health System, Danville, PA, USA; 2 Department of Vascular and Endovascular Surgery, Geisinger Health System, Danville, PA, USA; 3 Division of Cardiovascular Diseases, Mayo Clinic, Rochester, MN, USA; 4 Divisions of General Internal Medicine and Preventive Medicine, and the Center for Genetic Medicine, Feinberg School of Medicine, Northwestern University, Chicago, IL, USA; 5 Group Health Research Institute, Group Health Cooperative, Seattle, WA, USA; 6 Patient-Centered Research, Aurora Research Institute™, Aurora Sinai Medical Center, Milwaukee, WI, USA; 7 Department of Health Sciences Research, Mayo Clinic, Rochester, MN USA; 8 Department of Biomedical Informatics, Vanderbilt University, Nashville, TN, USA; 9 Biomedical Informatics Research Center, Marshfield Clinic Research Foundation, Marshfield, WI, USA; 10 The Charles Bronfman Institute for Personalized Medicine, Icahn School of Medicine at Mount Sinai, New York, NY, USA; 11 Essentia Institute of Rural Health, Duluth, MN, USA; 12 Departments of Medicine (Medical Genetics) and Genome Sciences, University of Washington, Seattle, WA, USA; 13 Cincinnati Children's Hospital Medical Center, Cincinnati, OH, USA; 14 Department of Surgery, Temple University School of Medicine, Philadelphia, PA, USA

**Keywords:** Electronic health records, Electronic medical record, Case-Control study, ICD-9, Computing methodologies, KNIME, Aortic aneurysm

## Abstract

**Background and objective:**

We designed an algorithm to identify abdominal aortic aneurysm cases and controls from electronic health records to be shared and executed within the “electronic Medical Records and Genomics” (eMERGE) Network.

**Materials and methods:**

Structured Query Language, was used to script the algorithm utilizing “Current Procedural Terminology” and “International Classification of Diseases” codes, with demographic and encounter data to classify individuals as case, control, or excluded. The algorithm was validated using blinded manual chart review at three eMERGE Network sites and one non-eMERGE Network site. Validation comprised evaluation of an equal number of predicted cases and controls selected at random from the algorithm predictions. After validation at the three eMERGE Network sites, the remaining eMERGE Network sites performed verification only. Finally, the algorithm was implemented as a workflow in the Konstanz Information Miner, which represented the logic graphically while retaining intermediate data for inspection at each node. The algorithm was configured to be independent of specific access to data and was exportable (without data) to other sites.

**Results:**

The algorithm demonstrated positive predictive values (PPV) of 92.8% (CI: 86.8-96.7) and 100% (CI: 97.0-100) for cases and controls, respectively. It performed well also outside the eMERGE Network. Implementation of the transportable executable algorithm as a Konstanz Information Miner workflow required much less effort than implementation from pseudo code, and ensured that the logic was as intended.

**Discussion and conclusion:**

This ePhenotyping algorithm identifies abdominal aortic aneurysm cases and controls from the electronic health record with high case and control PPV necessary for research purposes, can be disseminated easily, and applied to high-throughput genetic and other studies.

## Introduction

Electronic health records (EHRs) capture a large volume of clinical and physiologic data, and present a valuable resource for research. The “electronic Medical Records and Genomics” (eMERGE) Network was organized by the National Human Genome Research Institute (NHGRI) in 2007 to develop, disseminate, and apply approaches to combine DNA biorepositories with electronic medical record (EMR) systems for large-scale, high-throughput genetic research with the ultimate goal of returning genomic testing results to patients in a clinical care setting [1]. To accomplish these goals in the eMERGE Network an important first step is to develop robust algorithms, so called “ePhenotyping” tools, to identify cases and controls directly from the EHR for studies on specific diseases and traits [2-12]. eMERGE ePhenotypes are developed by one or more primary sites, validated at secondary sites and verified at all other sites that implement them. The results of this rigorous development effort are accurate, robust algorithms that may be used at other sites outside the eMERGE Network.

An abdominal aortic aneurysm (AAA) is a chronic progressively expanding dilatation of the abdominal aorta below the renal arteries and above the iliac artery bifurcation [13,14]. The Society of Vascular Surgery guidelines define an AAA as a dilatation greater than 3 cm in diameter. Most dilatations expand to exceed the threshold over time and there is an increased risk of rupture with catastrophic consequences when the diameter exceeds 5.5 cm [13,14].

In the present study we report the design of an ePhenotyping algorithm to identify cases with AAA and controls from the EHR. Structured Query Language (SQL) was used to script the algorithm utilizing “Current Procedural Terminology” (CPT) and “International Classification of Diseases” (ICD-9) codes as well as demographic and encounter data to classify individuals as case, control, or excluded. The algorithm was validated on a subset of individuals by manual chart review, and then implemented at six other eMERGE network sites and one site not part of the network. Finally, the algorithm was implemented as a workflow in the Konstanz Information Miner (KNIME) (http://www.knime.org/) [15,16].

## Materials and Methods

Seven eMERGE Network institutions and Aurora Health System participated in this study (Table 1). All institutions obtained appropriate approval from their respective institutional review boards, and made use of a common data use agreement to enable data sharing between institutions [1]. Each institution used an EHR for documentation of routine clinical care linked to a research specimen biorepository. Each site's cohort of genotyped individuals are targeted for specific diseases and mixed with an appropriate number of controls (Table 1).

### Development and validation of an ePhenotyping algorithm for AAA

**Figure 1:**
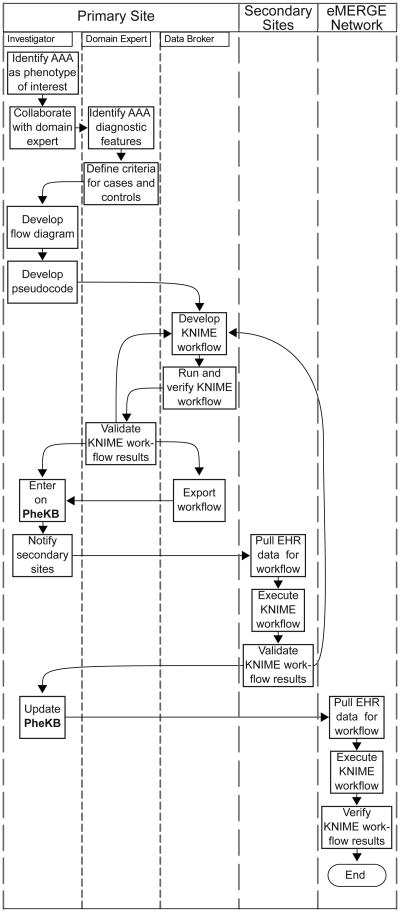
Overview of ePhenotyping. The diagram outlines the processes for phenotype algorithm development, validation and implementation as a KNIME workflow, as well as the interactions between the various study sites and investigators outside the eMERGE Network. AAA, abdominal aortic aneurysm; EHR, Electronic Health Record; eMERGE, electronic MEdical Records and GEnomics Network (http://www.gwas.org); KNIME, Konstanz Information Miner (http://www.knime.org/); PheKB, Phenotype KnowledgeBase available at http://www.phekb.org, an online collaborative repository for building, validating, and sharing electronic phenotype algorithms and their performance characteristics.

**Figure 2:**
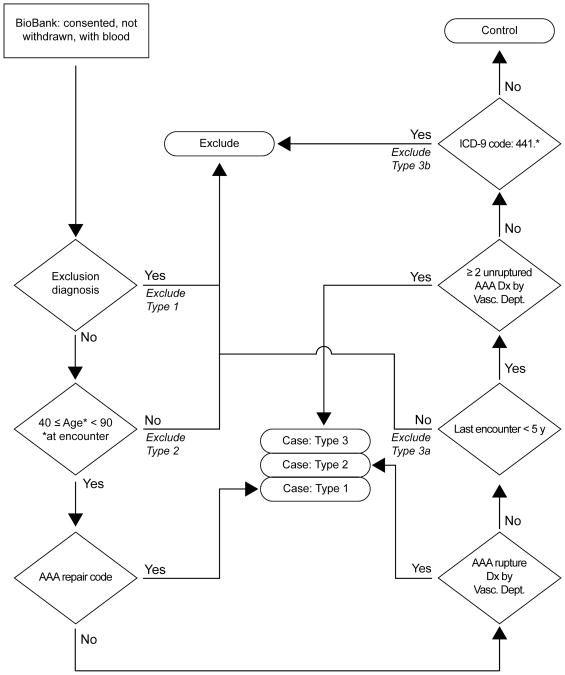
ePhenotyping algorithm for the identification of cases with abdominal aortic aneurysms (AAA) and appropriate controls for research studies. For codes used in the algorithm, see Supplementary Material Table 1.

Figure 1 outlines the development process and validation logic for the AAA ePhenotyping algorithm. SQL was used to script the algorithm utilizing CPT and ICD-9 codes (Supplementary Material Table 1) as well as demographic and encounter data to classify individuals as case, control, or excluded. AAA cases were defined as having an AAA repair procedure (case Type 1), or at least one appropriate specialty encounter (vascular clinic) with a ruptured AAA (case Type 2), or at least two specialty encounters with an unruptured AAA (case Type 3) (Figure 2). Controls were neither cases nor those excluded, had an encounter within the past 5 years, and had never been assigned an ICD9 code of 441.*, where * is a 1 or 2 digit code. Individuals were excluded if 1) they had a rare heritable disease with aortic manifestation or thoracic aortic aneurysm (Supplementary Material Table 1); 2) they were younger than 40 or older than 89 years, 3) they had a single encounter with a code without mention of rupture (441.4), or 4) they had not had an encounter within the past 5 years. Rare heritable diseases were excluded because the goal of the current study was to identify non-syndromic AAA [13,14]. Controls under 40 years might yet manifest an AAA, while cases under 40 years of age and without rare syndromic forms of aortic aneurysms are likely due to trauma. The full list of all CPT and ICD-9 codes used in the algorithm is provided in Supplementary Material Table 1. The AAA algorithm can be downloaded from www.PheKb.org.

### Evaluation of algorithm performance

Two different types of evaluation of the performance of the algorithm were carried out, namely verification and validation. Verification consisted in ensuring that the algorithm correctly identified cases, controls and those excluded by performing chart review knowing the algorithm call for a specific individual. Verification was used during algorithm development (Figure 1). Validation consisted in performing chart review blinded to the algorithm call. The expert opinions of trained chart reviewers or domain experts were then used to evaluate case positive predictive value (PPV), and control PPV. The standard formula for PPV was used with the gold standard defined as the expert opinion and test outcome considered to be the algorithm call.

### Implementation in KNIME

**Figure 3:**
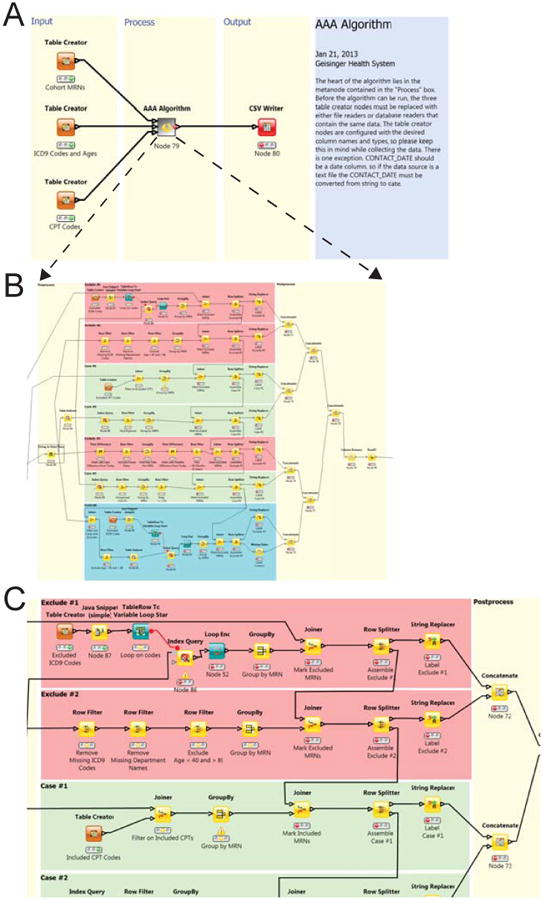
KNIME representation of the abdominal aortic aneurysm (AAA) ePhenotyping algorithm. (A) Global overview of the algorithm. The inputs on the left are abstract representations of the data required by the algorithm. The data fields are enumerated in each “Table Creator” node. Each site can supply the input data via any of KNIME's data reader nodes, provided all fields are present, named according to the templates and of the correct data type. The algorithm is encapsulated in the central meta node. (B) Expansion of the meta node, showing individual KNIME nodes with annotation and graphic background to facilitate comprehension of algorithm steps. (C) Enlarged top portion of (B) for readability.

**Figure 4:**
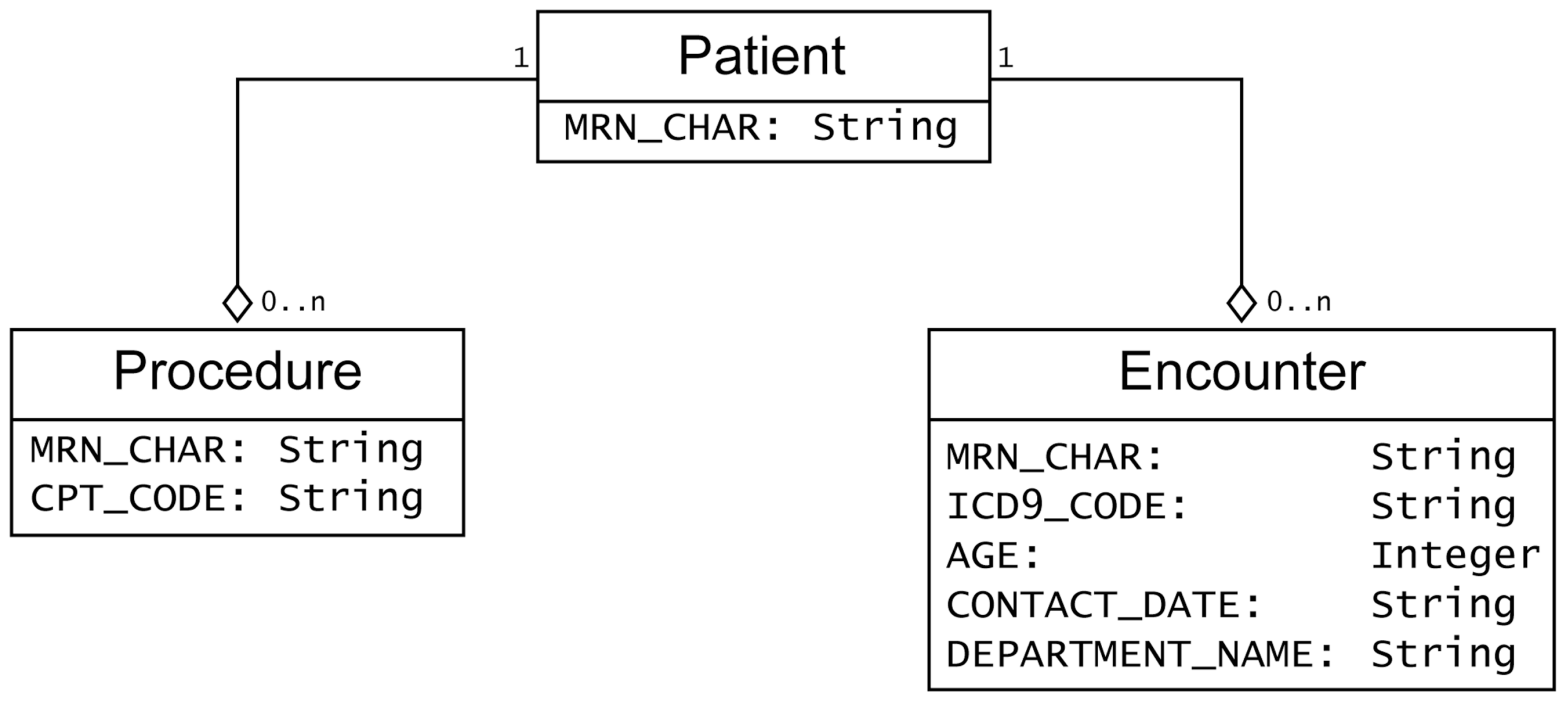
Entity relational diagram (ERD) of the input for the AAA ePhenotyping algorithm.

KNIME is an open source integrated development environment (IDE) designed to facilitate drag-and-drop, graphical programming workflows [15-19]. Each workflow consists of many atomic functional units called nodes. Nodes complete a single, specific task, such as sorting a list or extracting some text. Execution can be initiated from any node, which will cause all prior nodes to execute. When a node is finished executing, that node's child will be executed next. If a node has more than one child, the children will be executed in parallel provided they are not dependent on some other process. The AAA workflow was divided into three sections: data load, phenotype processing, and output of results (Figure 3). The data load (Figure 4) and output sections were designed to be abstractions that could easily be customized by sites. The phenotype processing section utilizes filters, full text search, and aggregating nodes to isolate each group of cases, controls, and excluded individuals. Finally, each group is labeled, and the groups merged.

The interface for loading data into the algorithm was abstracted in that only the fields and their data types were specified, not the precise mechanism of obtaining the data from the local repository. Thus the data could be accessed by any number of I/O nodes provided by KNIME: direct SQL access to databases via database connector and database reader nodes, or from exported files in standard formats such as tab-delimited, comma separated variable or XML formats. Abstraction allows local sites to determine the mechanism of data access that conform to their rules and regulations. The AAA algorithm is based on use of ICD-9 and CPT codes as well as encounter data. Three streams of data were specified: 1) identifiers, consisting of the cohort subject identifiers; 2) ICD-9 codes, consisting of subject identifiers, the date when the code was assigned, the ICD-9 code, the clinic where the code was assigned, and the age of the subject at the time; and 3) CPT codes, consisting of subject identifiers, the date when the code was assigned, the CPT code, the clinic where the code was assigned, and the age of the subject at the time. The algorithm filtered the ICD-9 and CPT code streams to restrict subsequent steps using the cohort identifiers. The identifier stream and filtering step could be redundant if the ICD-9 and CPT streams of data were provided as pre-filtered extracted files, but permitted the maximal flexibility of data input.

## Results and Discussion

eMERGE ePhenotyping algorithms are developed using an iterative approach where one of the sites, the primary site, proposes an algorithm and develops the first iteration [6]. One or more secondary sites then implement the algorithm and perform validation (Figure 1). If the algorithm performs poorly or the algorithm is difficult to implement, the primary site revises the algorithm using the comments from the secondary site and the process is repeated, until the algorithm performs to a desired criteria at both primary and secondary sites. Each iteration involves blinded validation. The AAA phenotype was proposed by the primary site Geisinger Health System (GHS), with Marshfield Clinic and Mayo Clinic acting as secondary sites. The algorithm was also implemented and validated at Aurora Health System, a site that is not a member of the eMERGE Network, demonstrating generalizability of the eMERGE algorithms.

### Establishment of diagnostic criteria

The objective of the study was to identify individuals with AAA not due to trauma or rare heritable diseases for genetic studies [13,14]. At GHS, the domain experts on AAA are the vascular surgeons; working closely together we elucidated their workflow and defined criteria for determining individuals who are suspected of having, or have, an AAA. Equally important was defining the criteria for excluding individuals who had AAAs due to identifiable conditions that would be phenocopies, i.e., individuals who had the same phenotype, but also had other aortic involvement such as thoracic aneurysm or dissection.

Three types of cases were defined in order of decreasing certainty: Type 1: individuals with a CPT code for AAA repair; Type 2: individuals who had an ICD-9 code for ruptured AAA; and, Type 3: individuals who had two ICD-9 codes on separate dates in one or more specialty clinics tasked with the diagnosis or treatment of AAA at the healthcare site, e.g., vascular surgery at GHS. Single and non-specialty clinic encounters were excluded because of the likelihood of a false-positive due to preliminary, unconfirmed diagnosis or miscoding.

### Development of the algorithm flowchart

Criteria and flow logic were developed iteratively and formalized into a flowchart (Figure 2). Candidate EMR records were identified by searching for the ICD-9 codes for AAA (441.3 and 441.4). Individuals with known rare heritable disorders with aneurysm manifestations such as the Marfan syndrome (ICD-9 759.82), Ehlers-Danlos syndrome (ICD-9 756.83), Moyamoya disease (ICD-9 437.5) or fibromuscular dysplasia (ICD-9 447.8), were excluded (Supplementary Material Table 1). In addition, we excluded individuals with diagnoses of other aortic aneurysms or aortic ruptures, or of generalized arterial dissections (Supplementary Material Table 1). Although not an exhaustive list of phenocopies, these comprise the most prevalent disorders and are also the ones with ICD-9 codes. We also excluded anyone with an AAA code at the age of 40 or younger since these are likely due to some pathogenic mechanism other than that of most AAAs, such as trauma or an unrecognized syndromic form.

Individuals over 40 years of age with repair codes for AAA (Supplementary Material Table 1) were identified as Type 1 cases. Next, individuals with ICD-9 codes for ruptured AAA (444.3) were identified as Type 2 cases. Among the remaining individuals, records were searched for the presence of two or more AAA diagnosis codes assigned on different visits at an appropriate clinic (vascular surgery at GHS) and the individuals identified as Type 3 cases. The requirement for 2 or more diagnosis codes was to ensure that working hypothesis diagnoses or referral diagnoses, that were not confirmed, were excluded.

After excluding phenocopies and young individuals, and identifying the cases, the remaining individuals were subjected to a series of filters designed to ensure that controls have a low probability of having AAA. Since AAA is a late age-at-onset disease [13,14], it is nearly impossible to ensure that controls truly will never have an AAA. First, we required that individuals had at least 5 years of follow-up in the EMR to increase the likelihood that occult AAAs could be detected. Finally, individuals who had any remaining ICD-9 codes related to aortic aneurysm, including single occurrences of ICD-9 for AAA, were excluded. Individuals who passed all exclusion filters and were not AAA cases were assigned control status (Figure 2). In genetic studies it is important to make sure that cases are true cases and controls are free of the disease under study. The filters used here provided a stringent mechanism for identifying true cases and controls and yielded a better study group than using diagnosis and AAA repair procedure codes alone.

### Performance of the algorithm

The algorithm was developed as part of the eMERGE Network activity, which emphasizes the integration of genomic data with EMRs. GHS contributed 3,111 individuals who were genotyped using high-density genotyping arrays to the collection of eMERGE genotyped biobank individuals (Table 1). To identify AAA cases and controls for genetic analyses we restricted the search to these 3,111 individuals and found 699 cases and 1,591 controls. There were 171 individuals that were excluded due to having known rare heritable disorders with aneurysm manifestation, or with thoracic or thoracoabdominal aortic aneurysms. The age criterion (younger than 40) excluded 559 individuals. Only 31 individuals were excluded due to no follow-up visits within the health system (5-year follow-up criterion) and 60 individuals were excluded due to not having been seen at a specialty clinic. The algorithm was validated on a randomly selected subset of individuals (n=100; 50 predicted cases and 50 predicted controls) by 2-fold manual chart review (each chart was reviewed by two independent reviewers) and demonstrated a case PPV of 94% and control PPV of 100% (Table 2). Among the 50 charts of predicted cases reviewed there were three false positives among the AAA cases identified by the algorithm. Two of these false positives were initially diagnosed as AAA as a result of having an abdominal aortic diameter of greater than 3 cm on an ultrasound, but were later changed after a computerized tomography (CT) scan showed that the size was actually less than 3 cm; these individuals had ectactic aortae and could be considered pre-aneurysmal. The other false positive was a complex aneurysm that was mostly in the ileac artery, but coded with both the 442.4 and 441.4 ICD-9 codes.

We also applied the AAA algorithm to the EMRs of all individuals consented for the GHS MyCode™ biobank. At the time of execution, the biobank comprised 29,770 individuals, and the algorithm identified 1,155 AAA cases and 17,523 controls. We excluded 337 individuals based on predisposing genetic conditions, 109 individuals without a visit within the past 5 years and 10,398 individuals based on age.

The algorithm was then tested independently at the Marshfield Clinic, which uses an internally developed CattailsMD EMR system, and Mayo Clinic, which uses GE Centricity, and implemented at four other eMERGE Network sites. At Marshfield Clinic the case PPV was 100% and control PPV was 100% indicating that there were no false positives in the case or control groups (Table 2). At Mayo Clinic the case and control PPV were 88% and 100%, respectively. At Mayo Clinic there were 6 false positives among the 50 cases reviewed manually (Table 2). Most of the false positive cases had AAA diagnosis codes, with a dilated abdominal aorta (aortic ectasia), but the size did not meet the formal definition of AAA (>3 cm) [13,20]. Prior studies suggest that many, if not most, individuals with dilated abdominal aortas subsequently develop AAA [21]. The algorithm therefore detected “pre-aneurysmal manifestations.”

Finally, the algorithm was implemented and validated at Aurora Health System, which is not a member of the eMERGE Network (Table 2). The algorithm performed very well at Aurora Health System achieving the case and control PPV of 96 and 100, respectively. There were 2 false positives among the 50 cases reviewed manually (Table 2). One of them had a popliteal artery aneurysm and the other patient had a pre-aneurysmal aortic dilatation, which was not confirmed in subsequent imaging studies using computerized tomography.

Overall, the algorithm demonstrated PPV of 92.8% (CI: 86.8–96.7) and 100% (CI: 97.0–100) for cases and controls, respectively.

The counts for identified AAA cases and controls using the algorithm at eight different institutions are shown in Table 1, and the validation results are summarized in Table 2. In total, there were 1,490 cases and 38,393 controls with biobanked blood samples. Of these 1,234 cases and 22,382 controls already have genome-wide data available for genetic studies.

We next looked at the distribution of AAA cases in the different case types in the three institutions with the largest number of AAA cases (Table 3) and how well the algorithm performed in identifying them. As can be seen in Table 3 at GHS, a substantial proportion of cases were Type 1 cases with AAA repair codes (42.2%), whereas at Aurora Health System, the majority of the cases were of Type 3 (97.3%). Table 4 shows a confusion matrix depicting the categorization of results from these three sites. Most false positives detected during the validation process were erroneously categorized as “case, Type 3.” These were caused by cases that required at least two visits to the vascular clinic before they were diagnosed as not having an AAA. It is possible that these false positives could be addressed by adding natural language processing (NLP) to determine the aneurysm size.

Each eMERGE site implements an ePhenotyping algorithm on a cohort of individuals that are targeted for certain diseases. The GHS cohort was selected for AAA and obesity; therefore it may not be surprising that we achieved a high PPV. However, by validating the algorithm at Mayo and Marshfield Clinics, which have cohorts that are targeted for cardiovascular and eye diseases, respectively, and achieving similar results shows that the algorithm performs well in other biobanks. Furthermore, by achieving excellent results at Aurora Health System, whose cohort is not selected for any specific disease, we demonstrate that the algorithm is universally applicable.

### KNIME workflow implementation to improve transportability of the ePhenotyping algorithm

Transportability must be considered when designing an ePhenotyping algorithm that is meant to be executed at different clinical sites. Each potential site may have a different EMR system with different access mechanisms and names for data objects, therefore algorithms must be shared in a generic and portable way. Typically, eMERGE sites exchange algorithm pseudocode, allowing each site to implement the algorithm as needed for its environment. This can be very time and labor intensive, and may produce unintended results if the pseudocode is interpreted incorrectly.

As an alternative to this practice, we determined the feasibility of using a portable executable algorithm in the form of a KNIME workflow. A KNIME workflow can be exported in a generic way (without data or site-specific identifiers), and easily imported into a KNIME environment at another site. Each site must extract and transform data from its EHR data repository so that it conforms to KNIME workflow specifications, allowing the algorithm to be executed with little to no customization (Figures 1 and 3). This helps to ensure that the logic of the algorithm is unaltered from its original state, therefore eliminating unintended results based on misinterpretation of pseudocode.

The eMERGE group at Northwestern University tested the remote implementation of the KNIME workflow for the AAA ePhenotype in their Epic EMR system. Although they found it to be easy to implement, they had to make a minor adjustment to the KNIME workflow to make it compatible with their site. The algorithm requires Type 2 and Type 3 cases to have been seen by a specialty clinic that is responsible for diagnosing AAA. For GHS, this specialty clinic is the vascular clinic, and the KNIME workflow was preset to allow for this requirement. At Northwestern University, the responsible clinics also included cardiology and interventional radiology, so the workflow had to be edited to accept this difference. This could have been overcome by designing the KNIME workflow to allow the responsible clinic to be entered as a variable.

Implementation of the algorithm at other sites was not technically demanding, but effective implementation depended on knowledge of the clinical workflow at that institution. Tabulation of the ICD-9 codes by clinic type can prove useful in identifying the relevant clinic(s). Some sites had prior experience implementing algorithms that, like the AAA algorithm, relied predominantly on ICD-9 codes and were described in pseudocode and flowcharts. They found the KNIME implementation to be substantially less work. The most time-consuming aspect of implementation was validation or verification, i.e., manual checking of records to ensure that the algorithm performed correctly.

Other possible solutions to the problem of portability of ePhenotyping algorithms have been suggested in the literature. The Shared Health Research Information Network (SHRINE) tool [22] of the Informatics for Integrating Biology & the Bedside (i2b2), an NIH-funded National Center for Biomedical Computing, and the Virtual Data Warehouse (VDW) of the HMO Research Network (HMORN) [23] both offer the ability to share data with other members of a consortium by creating a federated database. Algorithms are then built to process the shared data, and can be distributed without preparation or alteration. Initial setup and maintenance of these databases have a high cost. All data must be transformed to an appropriate schema, loaded into the federated database, and maintained overtime. The expertise to perform such functions needs to be retained on each site's staff. ePhenotyping algorithms have also been distributed as National Quality Forum (NQF) Quality Data Model (QDM) [24] documents with certain levels of success [25]. Like federated databases, much preliminary work must be performed to create an infrastructure to allow for processing of these documents. We decided against these solutions in favor of reducing overhead and keeping project costs to a minimum.

## Conclusion

We designed a robust ePhenotyping algorithm and implemented it as a KNIME workflow to identify AAA cases and controls from the EMR with high case and control PPV necessary for research purposes. The resulting algorithm yields cases and controls with high confidence, since AAA due to trauma or rare heritable diseases, phenocopies, and working diagnoses are excluded. The KNIME workflow makes the algorithm easily transportable from one institution to another even with different EMR systems.

## Supplementary Material

Supplementary file

## Figures and Tables

**Table 1:** Case control counts and demographics at different sites.

	Biobank		AAA Cases				Controls			
Cohort	Participants	Target diseases†	N	Sex	Age§ (years)			Sex	Age§ (years)	
	N*			% Male	Mean	SD	N	% Male	Mean	SD
Aurora	17,902		256	80	73	10.04	16,011	44	63	15.37
GHS	3,111	AAA and obesity	699	81	71	7.43	1,591	47	62	10.86
Group Health	3,528	Alzheimer's disease	178	72	80	5.16	2,490	44	78	8.94
Marshfield	4,987	Eye diseases	100	71	74	8.48	3,928	38	72	10.46
Mayo	10,062	Cardiovascular diseases	178	81	73	7.65	4,930	54	67	10.90
Mount Sinai	6,545	Kidney diseases	30	94	76	7.58	5,324	41	63	11.98
Northwestern	4,937	Type 2 diabetes and cancer	11	73	65	6.98	3,967	17	54	14.60
Vanderbilt	9,584	Cardiovascular diseases	38	58	78	7.71	152	76	78	8.59
Total	60,656		1,490				38,393			

* For eMERGE sites the number of biobank participants is with high-density genome-wide data [1], and for Aurora, the number is the total number of consented patients with a blood sample in the biobank.

† Appropriate controls for the diseases mentioned were also included in the genome-wide data sets.

§ Age, age at diagnosis for cases, and age at enrollment for controls. AAA, Abdominal Aortic Aneurysm; GHS, Geisinger Health System.

**Table 2:** Summary of chart review results at four participating sites

	Manual chart review											
	GHS*			Mayo Clinic*			Marshfield Clinic*			Aurora Health†		
	Case	Control	Total	Case	Control	Total	Case	Control	Total	Case	Control	Total
EHR prediction												
Case	47	3	50	44	6	50	25	0	25	48	2	50
Control	0	50	50	0	50	50	0	22	22	0	50	50
Total	47	53	100	44	56	100	25	22	47	48	52	100
Case PPV			94			88			100			96
Control PPV			100			100			100			100

* Trained chart reviewers.

† Clinician chart reviewers.

GHS, Geisinger Health System; EHR, electronic health record.

**Table 3:** Distribution of AAA case types 1, 2 and 3 at GHS, Aurora Health System and Mayo Clinic biobanks.

Case Type	GHS		Aurora		Mayo	
	N	%	N	%	N	%
1	295	42.2	0	0	72	40.4
2	16	2.3	7	2.7	0	0
3	388	55.5	249	97.3	106	59.4
All	699	100	256	100	178	100

Breakdown of the AAA cases into those who were operated for AAA (case Type 1), who had a ruptured AAA (case Type 2), or who had at least twice an ICD-9 code for AAA in their EHR (case Type 3)

**Table 4:** Distribution of false positive AAA case types 1, 2 and 3 at GHS, Aurora Health System and Mayo Clinic based on manual chart review validation.

Case Type	GHS N	Aurora N	Mayo N	Total N
1	0	0	2	2
2	1	0	0	1
3	2	2	4	8
All	3	2	6	11

## References

[1] Gottesman O, Kuivaniemi H, Tromp G, Faucett WA, Li R (2013). The Electronic Medical Records and Genomics (eMERGE) Network: past, present, and future. Genet Med.

[2] Kho AN, Hayes MG, Rasmussen-Torvik L, Pacheco JA, Thompson WK (2012). Use of diverse electronic medical record systems to identify genetic risk for type 2 diabetes within a genome-wide association study. J Am Med Inform Assoc.

[3] Kho AN, Pacheco JA, Peissig PL, Rasmussen L, Newton KM (2011). Electronic medical records for genetic research: results of the eMERGE consortium. Sci Transl Med.

[4] Kho AN, Rasmussen LV, Connolly JJ, Peissig PL, Starren J (2013). Practical challenges in integrating genomic data into the electronic health record. Genet Med.

[5] Muthalagu A, Pacheco JA, Aufox S, Peissig PL, Fuehrer JT (2014). A rigorous algorithm to detect and clean inaccurate adult height records within EHR systems. Appl Clin Inform.

[6] Newton KM, Peissig PL, Kho AN, Bielinski SJ, Berg RL (2013). Validation of electronic medical record-based phenotyping algorithms: results and lessons learned from the eMERGE network. J Am Med Inform Assoc.

[7] Pacheco JA, Avila PC, Thompson JA, Law M, Quraishi JA (2009). A highly specific algorithm for identifying asthma cases and controls for genome-wide association studies. AMIA Annu Symp Proc.

[8] Peissig PL, Rasmussen LV, Berg RL, Linneman JG, McCarty CA (2012). Importance of multi-modal approaches to effectively identify cataract cases from electronic health records. J Am Med Inform Assoc.

[9] Rasmussen LV, Peissig PL, McCarty CA, Starren J (2012). Development of an optical character recognition pipeline for handwritten form fields from an electronic health record. J Am Med Inform Assoc.

[10] Rasmussen LV, Thompson WK, Pacheco JA, Kho AN, Carrell DS (2014). Design patterns for the development of electronic health record-driven phenotype extraction algorithms. J Biomed Inform.

[11] Waudby CJ, Berg RL, Linneman JG, Rasmussen LV, Peissig PL (2011). Cataract research using electronic health records. BMC Ophthalmol.

[12] Wei WQ, Feng Q, Jiang L, Waitara MS, Iwuchukwu OF (2014). Characterization of statin dose response in electronic medical records. Clin Pharmacol Ther.

[13] Lederle FA (2009). In the clinic. Abdominal aortic aneurysm. Ann Intern Med.

[14] Kent KC (2014). Clinical practice. Abdominal aortic aneurysms. N Engl J Med.

[15] Silipo R (2011). KNIME Beginner's Luck.

[16] Silipo R, Mazanetz MP (2012). The KNIME Cookbook.

[17] Beisken S, Meinl T, Wiswedel B, de Figueiredo LF, Berthold M (2013). KNIME-CDK: Workflow-driven cheminformatics. BMC Bioinformatics.

[18] Jagla B, Wiswedel B, Coppée JY (2011). Extending KNIME for next-generation sequencing data analysis. Bioinformatics.

[R19] 19http://www.knime.com/2014-08-31

[20] Bohlin S, Fröjd C, Wanhainen A, Björck M (2014). Change in smoking habits after having been screened for abdominal aortic aneurysm. Eur J Vasc Endovasc Surg.

[21] d'Audiffret A, Santilli S, Tretinyak A, Roethle S (2002). Fate of the ectatic infrarenal aorta: expansion rates and outcomes. Ann Vasc Surg.

[22] Weber GM, Murphy SN, McMurry AJ, Macfadden D, Nigrin DJ (2009). The Shared Health Research Information Network (SHRINE): a prototype federated query tool for clinical data repositories. J Am Med Inform Assoc.

[23] Ross TR, Ng D, Brown JS, Pardee R, Hornbrook MC (2014). The HMO Research Network Virtual Data Warehouse: A Public Data Model to Support Collaboration. EGEMS (Wash DC).

[24] Li D, Endle CM, Murthy S, Stancl C, Suesse D (2012). Modeling and executing electronic health records driven phenotyping algorithms using the NQF Quality Data Model and JBoss® Drools Engine. AMIA Annu Symp Proc.

[25] Thompson WK, Rasmussen LV, Pacheco JA, Peissig PL, Denny JC (2012). An evaluation of the NQF Quality Data Model for representing Electronic Health Record driven phenotyping algorithms. AMIA Annu Symp Proc.

