# Supplementary material for: ePhenotyping for Abdominal Aortic Aneurysm in the Electronic Medical Records and Genomics (eMERGE) Network: Algorithm Development and Konstanz Information Miner Workflow
Source: Int J Biomed Data Min. Author manuscript; Available in PMC 2016 Apr 4. (PMC4820287)
Supplement: Supplementary file [file NIHMS723318-supplement-Supplementary_file.docx]

**Appendix 1. Supplementary data**

**SUPPLEMENTARY MATERIAL**

**Table 1 —** CPT and ICD-9 codes used in the algorithm

| **CPT code Description** | |
| --- | --- |
| 34800 | Endovascular repair of infrarenal abdominal aortic aneurysm or dissection; using aorto-aortic tube prosthesis |
| 34802 | Endovascular repair of infrarenal abdominal aortic aneurysm or dissection; using modular bifurcated prosthesis (1 docking limb) |
| 34803 | Endovascular repair of infrarenal abdominal aortic aneurysm or dissection; using modular bifurcated prosthesis (2 docking limbs) |
| 34804 | Endovascular repair of infrarenal abdominal aortic aneurysm or dissection; using unibody bifurcated prosthesis |
| 34805 | Endovascular repair of infrarenal abdominal aortic aneurysm or dissection; using aorto-uniiliac or aorto‐unifemoral prosthesis |
| 34830 | Open repair of infrarenal aortic aneurysm or dissection, plus repair of associated arterial trauma, following unsuccessful endovascular repair; tube prosthesis |
| 34831 | Open repair of infrarenal aortic aneurysm or dissection, plus repair of associated arterial trauma, following unsuccessful endovascular repair; aorto‐bi‐iliac prosthesis |
| 34832 | Open repair of infrarenal aortic aneurysm or dissection, plus repair of associated arterial trauma, following unsuccessful endovascular repair; aorto‐bifemoral prosthesis |
| 35081 | Direct repair of aneurysm, pseudoaneurysm, or excision (partial or total) and graft insertion, with or without patch graft; for aneurysm, pseudoaneurysm, and associated occlusive disease, abdominal aorta |
| 35082 | Direct repair of aneurysm, pseudoaneurysm, or excision (partial or total) and graft insertion, with or without patch graft; for ruptured aneurysm, abdominal aorta |
| 35091 | Direct repair of aneurysm, pseudoaneurysm, or excision (partial or total) and graft insertion, with or without patch graft; for aneurysm, pseudoaneurysm, and associated occlusive disease, abdominal aorta involving visceral vessels (mesenteric, celiac, renal) |
| 35092 | Direct repair of aneurysm, pseudoaneurysm, or excision (partial or total) and graft insertion, with or without patch graft; for ruptured aneurysm, abdominal aorta involving visceral vessels (mesenteric, celiac, renal) |
| 35102 | Direct repair of aneurysm, pseudoaneurysm, or excision (partial or total) and graft insertion, with or without patch graft; for aneurysm, pseudoaneurysm, and associated occlusive disease, abdominal aorta involving iliac vessels (common, hypogastric, external) |
| 35103 | Direct repair of aneurysm, pseudoaneurysm, or excision (partial or total) and graft insertion, with or without patch graft; for ruptured aneurysm, abdominal aorta involving iliac vessels (common, hypogastric, external) |
| 35131 | Direct repair of aneurysm, pseudoaneurysm, or excision (partial or total) and graft insertion, with or without patch graft; for aneurysm, pseudoaneurysm, and associated occlusive disease, iliac artery (common, hypogastric, external) |
| 35132 | Direct repair of aneurysm, pseudoaneurysm, or excision (partial or total) and graft insertion, with or without patch graft; for ruptured aneurysm, iliac artery (common, hypogastric, external) |
|  |  |
| **ICD-9 codes for inclusion** | |
| 441.3 | Abdominal aortic aneurysm, ruptured |
| 441.4 | Abdominal aortic aneurysm, without mention of rupture |
| 441.9 | Aortic aneurysm of unspecified site without mention of rupture |
|  |  |
| **ICD-9 codes for exclusion** | |
| 759.82 | Marfan Syndrome |
| 756.83 | Ehlers‐Danlos Syndrome |
| 441.1 | Thoracic Aneurysm, ruptured |
| 441.2 | Thoracic Aneurysm without mention of rupture |
| 441.7 | Thoracoabdominal aneurysm without mention of rupture |
| 443.2 | Other arterial dissection |
| 437.5 | Moyamoya Disease |
| 447.8 | Fibromuscular Dysplasia |

**Supplementary Form 1: Geisinger Clinic Abdominal Aortic Aneurysm Abstraction Form (eMERGE)**

**Site __ __ __ __ eMERGE ID __ __ __ __ Recruitment date __ __ / __ __ / __ __ __ __ (MM/DD/YYYY)**

**Reviewer ID __ __ Reviewed Date __ __ / __ __ / __ __ __ __ (MM/DD/YYYY)**

**AAA Status: 🞎 AAA 🞎 Probable AAA 🞎 No AAA 🞎 Exclude**

**Section 1: Has the patient had an encounter within the past five (5) years?**

- **No. Go to Section 2**
- Yes. **STOP and classify as “Exclude”.**

**Section 2: Does the patient have a rare genetic disease?**

- Marfan syndrome ICD9 759.82
- Ehlers-Danlos syndrome ICD9 756.83
- Loeys-Dietz syndrome
- Thoracic aortic aneurysm and dissection ICD9 441.1, 441.2, or 443.2
- Moyamoya disease ICD9 437.5
- Fibromuscular dysplasia ICD9 447.8

**If YES to ANY question in 2, STOP and classify as “Exclude”;**

**If NO to ALL questions in 2, continue to Section 3.**

**Section 3:**

**3a: Endovascular repair of AAA?**

- **No. Go to Section 3b**
- Yes. Choose the 1^st^ procedure, **STOP and classify as “AAA”.**

__ __ / __ __ / __ __ __ __ (MM/DD/YYYY)

**3b: Open repair of AAA?**

- No. **Go to Section 4**
- Yes. Choose the 1st procedure **STOP and classify as “AAA”**

__ __ / __ __ / __ __ __ __ (MM/DD/YYYY)

**Section 4: The patient has a diagnosis of a ruptured AAA with code**

- Abdominal aneurysm, ruptured, ICD9 441.3

**If YES to ANY question in 4, STOP and classify as “AAA”;**

**If NO to ALL questions in 4, continue to Section 5.**

**Section 5: A diagnosis of AAA confirmed by a Vascular Surgeon at a Vascular Surgery (or appropriate specialty) Department?**

- Abdominal aneurysm without mention of rupture, ICD9 441.4
- Aortic aneurysm of unspecified site without mention of rupture, ICD9 441.9

**If YES to ANY of the diagnoses, STOP and classify as “AAA”**

**If NO to ALL questions in 5, continue to Section 6.**

**Section 6: A diagnosis of AAA originating in a department other than Vascular Surgery or appropriate specialty?**

- **Yes. STOP and classify as “Exclude”.**

No. Continue to Section 7.

**Section 7: Abdominal Imaging Procedure?**

- **No. STOP and classify as “No AAA”.**
- Yes. Choose the most recent test with normal result or the 1^st^ test with abnormal result (mark any of the below)

**Type of Report:**

- Magnetic Resonance Angiography (MRA) __ __ /__ __ /__ __ __ __(MM/DD/YYYY)
- Computed Tomographic Angiography (CTA) __ __ /__ __ /__ __ __ __ (MM/DD/YYYY)
- Abdominal Ultrasound __ __ /__ __ /__ __ __ __ (MM/DD/YYYY)
- Aortogram with runoff __ __ /__ __ /__ __ __ __(MM/DD/YYYY)
- Other ________________________ __ __ /__ __ /__ __ __ __(MM/DD/YYYY)

Any mention of dilatation/aneurysm in the imaging report?

- Yes. Check size and location

Size: ____________ cm

Location: ______________

- No.

**“Probable AAA”** if: infrarenal aortic diameter > 3 cm

**“No AAA”** infrarenal aortic diameter < 3 cm
